# Sequential levetiracetam and phenytoin in electroencephalographic neonatal seizures unresponsive to phenobarbital: a multicenter prospective observational study in India

**DOI:** 10.1016/j.lansea.2024.100371

**Published:** 2024-02-15

**Authors:** Vaisakh Krishnan, Vidya Ujjanappa, Hemadri Vegda, Manjesh K. Annayappa, Pooja Wali, Sudhindrashayana Fattepur, Savitha Chandriah, Sahana Devadas, Mallesh Kariappa, Veluthedath Kuzhiyil Gireeshan, Ajithkumar Vellani Thamunni, Paolo Montaldo, Constance Burgod, Reema Garegrat, Pallavi Muraleedharan, Stuti Pant, Charles R. Newton, J Helen Cross, Paul Bassett, Seetha Shankaran, Sudhin Thayyil, Ronit M. Pressler

**Affiliations:** a Centre for Perinatal Neuroscience, Imperial College, London, United Kingdom; b Department of Pediatrics, Karnataka Institute of Medical Sciences, Hubballi, India; c Department of Obstetrics and Gynecology, Bangalore Medical College and Research Institute, Bengaluru, India; d Department of Pediatrics, Bangalore Medical College and Research Institute, Bengaluru, India; e Department of Pediatrics, Government Medical College, Kozhikode, India; f Department of Neonatology, Università Degli Studi della Campania Luigi Vanvitelli, Naples, Italy; g Department of Psychiatry, University of Oxford, United Kingdom; h UCL Great Ormond Street Institute of Child Health & Great Ormond Street Hospital for Children, London, United Kingdom; i Statsconsultancy Ltd, Amersham, United Kingdom; j Department of Neonatal-Perinatal Medicine, Wayne State University, Detroit, MI, USA; k Department of Neurophysiology, Great Ormond Street Hospital, United Kingdom; l University of Texas at Austin, Dell Children’s Hospital, Austin, USA; m Department of Clinical Neuroscience, UCL-Great Ormond Street Institute of Child Health, London, United Kingdom

**Keywords:** Neonatal seizures, Levetiracetam, Phenytoin, Epilepsy

## Abstract

**Background:**

Although levetiracetam and phenytoin are widely used antiseizure medications (ASM) in neonates, their efficacy on seizure freedom is unclear. We evaluated electroencephalographic (EEG) seizure freedom following sequential levetiracetam and phenytoin in neonatal seizures unresponsive to phenobarbital.

**Methods:**

We recruited neonates born ≥35 weeks and aged <72 h who had continued electrographic seizures despite phenobarbital, from three Indian hospitals, between 20 June 2020 and 31 July 2022. The neonates were treated with intravenous levetiracetam (20 mg/kg x 2 doses, second line) followed by phenytoin (20 mg/kg x 2 doses, third line) if seizures persisted. The primary outcome was complete seizure freedom, defined as an absence of seizures on EEG for at least 60 min within 40 min from the start of infusion.

**Findings:**

Of the 206 neonates with continued seizures despite phenobarbital, 152 received levetiracetam with EEG. Of these one EEG was missing, 47 (31.1%) were in status epilepticus, and primary outcome data were available in 145. Seizure freedom occurred in 20 (13.8%; 95% CI 8.6%–20.5%) after levetiracetam; 16 (80.0%) responded to the first dose and 4 (20.0%) to the second dose. Of the 125 neonates with persisting seizures after levetiracetam, 114 received phenytoin under EEG monitoring. Of these, the primary outcome data were available in 104. Seizure freedom occurred in 59 (56.7%; 95% CI 46.7%–66.4%) neonates; 54 (91.5%) responded to the first dose and 5 (8.5%) to the second dose.

**Interpretation:**

With the conventional doses, levetiracetam was associated with immediate EEG seizure cessation in only 14% of phenobarbital unresponsive neonatal seizures. Additional treatment with phenytoin along with levetiracetam attained seizure freedom in further 57%. Safety and efficacy of higher doses of levetiracetam should be evaluated in well-designed randomised controlled trials.

**Funding:**

10.13039/501100000272National Institute for Health and Care Research (NIHR) Research and Innovation for Global Health Transformation (NIHR200144).

**Research in context**

**Evidence before this study**

The international league against epilepsy taskforce recommends phenobarbital as first line treatment for neonatal seizures. However, no definite recommendations are available for second line antiseizure medications due to lack of evidence.

Prior to the current study, we searched PubMed (January 1985 to June 2020) using the key words “neonates” OR “newborn” OR “infant” AND “levetiracetam” OR “keppra” OR “phenytoin” AND “seizures” OR “convulsions” OR “fits” OR “epileptiform discharges” to identify prospective studies (observational, quasi randomised or randomised control trials) evaluating the efficacy of levetiracetam and/or phenytoin on electroencephalographic (EEG) in neonatal seizures unresponsive to phenobarbital. Following completion of the study, we updated the search in April 2023. We did not identify any prospective studies evaluating efficacy of levetiracetam or phenytoin on EEG seizure freedom in neonatal seizures unresponsive to phenobarbital.

The available data on second line efficacy of levetiracetam with EEG in phenobarbital unresponsive neonatal seizures were from small subgroup in a retrospective review of 14 neonates and 6 neonates from a subgroup analysis within a randomized controlled trial comparing first line levetiracetam versus first line phenytoin.

**Added value of this study**

We report the first study from a low and middle-income country to use EEG monitoring in neonatal units to evaluate antiseizure medications. Of the 152 neonates with seizures unresponsive to phenobarbital, levetiracetam was associated with attainment of seizure freedom on EEG only in 14% of the neonates by 40 min from the start of the infusion. Further treatment with phenytoin along with levetiracetam resulted in seizure freedom in an additional 57% of the neonates. The increase in seizure freedom may be directly related to phenytoin, delayed effects of levetiracetam or synergy. Thirty one percent of the neonates were in status epilepticus indicating high disease severity.

**Implications of all the available evidence**

Although evidence from randomised controlled trials is lacking, the data from observational studies suggest that levetiracetam, in the standard doses, may not be an effective second line ASM for terminating neonatal seizures unresponsive to phenobarbital. Given the safety profile and potential neuroprotective effects in neonates, the effect of high dose levetiracetam on seizure cessation and neurodevelopmental outcome in LMIC should be explored in clinical trials.

## Introduction

Neonatal seizures are the most common neurological manifestation of brain injury in the neonatal period.^1^ Affected neonates die or survive with long-term neuro-disability and epilepsy.^2^ The burden of neonatal seizures in low- and middle-income countries (LMIC) is 10–30 times higher than in high-income countries.^3^^,^^4^

Clinical diagnosis of neonatal seizures is unreliable and subjective. Non seizures movements may be mis-interpreted as seizures leading to unnecessary treatment. Conversely, many electrographic seizures may not have clinical manifestations.^5^, ^6^, ^7^ Hence, medical regulatory bodies recommend that in clinical trials evaluating antiseizure medication (ASM), seizure freedom should be examined using EEG.^8^, ^9^, ^10^

As an ASM, levetiracetam has several advantages, particularly in LMIC due to its safety profile, minimal sedation, lack of respiratory suppression, ease of administration, favorable pharmacokinetics and potential neuroprotection.^11^^,^^12^ Pooled data from several small single centre open label randomised controlled trials from LMIC suggest levetiracetam has similar efficacy to phenobarbital in clinical seizure cessation (70% versus 56%).^13^, ^14^, ^15^ However, the efficacy of levetiracetam was much lower than phenobarbital (28% versus 80%) in a well-designed multicenter phase IIb randomised controlled trial where EEG was used to assess seizure cessation.^16^

We evaluated the efficacy of sequential levetiracetam and phenytoin administration in terminating neonatal seizures unresponsive to phenobarbital using continuous EEG monitoring in South India.

## Methods

### Study design and participants

We conducted a prospective multicenter observational study across three tertiary care public sector teaching hospitals in India (Karnataka Institute of Medical Sciences, Hubballi; Bangalore Medical College and Research Institute, Bengaluru; and Government Medical College, Kozhikode) between 20 June 2020 and 31 July 2022.

Neonates born at the recruiting hospital were defined as inborn, and the neonates born at other health care facilities or at home were defined as outborns. All inborn neonates born at or after 35 weeks of gestation and admitted to neonatal unit with encephalopathy or clinical seizures within 72 h after birth were screened for eligibility. Upon recognition of clinical or electrographic seizures, phenobarbital was administered as the first line ASM (a total of 30–40 mg/kg in 2 doses) if seizures continued after metabolic corrections.

We included all neonates who had continued seizures on EEG after 30 min of phenobarbital administration requiring additional ASM. The following neonates were excluded 1) outborn neonates 2) neonates who received second line ASM without EEG monitoring 3) transient metabolic disorders who responded to metabolic corrections and inborn errors of metabolism.

The study was approved by research ethics committees at Imperial College, London and the participating sites, and all parents provided written informed consent.

### Procedures

Prior to the study, the PREVENT (Prevention of Epilepsy by Reducing Neonatal Encephalopathy) research consortium was set up between Imperial College London, University College London, Oxford University and the three recruiting sites in India. A team of 6 neonatal neurology fellows, 6 neonatal research nurses, and 7 EEG technicians were appointed at the study sites in India and were trained and certified in various aspects of the study protocol including structured neurological assessment (modified Sarnat stage), EEG acquisition and interpretation. All sites were provided with Neurosoft-Neuron-Spectrum 4-P video EEG machines (Neurosoft LLC, Ivanovo, Russia), and were read with Neuron spectrum software version 2.0.22.1. The montage was based on the international 10–20 system modified for neonates with 13 electrodes. The video was recorded time-locked with the EEG and additional polygraphic channels included electrocardiography (ECG), respiratory effort, and bilateral surface electromyographic recordings (deltoid).

EEG recordings were commenced between 6 and 24 h after birth in neonates admitted with HIE ensuring peak seizure occurrence was captured. In neonates admitted with suspected seizures or encephalopathy unrelated to hypoxic ischaemic encephalopathy (HIE), EEG was started soon after admission. In all neonates, EEG was continued for at least 4 h if the recording was normal and up to 24 h if seizures were noted. The EEG reporting was undertaken by specialist neonatal neurology fellows (VK, VU and HV) at each site in real time under the supervision of an expert clinical neurophysiologist (RP) using a cloud-based real time EEG review system. All seizures were verified by two independent reviewers.

A seizure management protocol based on current evidence-based recommendations for LMIC^17^ was standardized across the sites and involved a step wise escalation starting with phenobarbital, followed by levetiracetam, then phenytoin, and finally midazolam. Neonates included in the study received levetiracetam (20 mg/kg) initially as a short infusion or slow iv push over 10–20 min and the dose was repeated if seizures persisted to achieve a maximal dose of 40 mg/kg. If seizures persisted despite maximal dose of levetiracetam, phenytoin (20 mg/kg) was administered as an infusion over 20–30 min and the dose was repeated (total 40 mg/kg) if seizures persisted. In between each infusion, a time gap of 10–20 min was given for the ASM to act unless the infant was in status epilepticus, where drug doses were escalated more rapidly.

Seizures were grouped into clinical events (no ictal EEG available), electrographic-only (EEG seizures without clinical manifestations), or electro-clinical seizures (EEG seizures with a clinical correlate).^4^ Diagnostic certainty of seizures was documented as defined by the Brighton Collaboration Neonatal Seizures Working Group.^4^^,^^18^ Level 1 included seizures confirmed with EEG, level 2 included clinical focal clonic or tonic seizures or seizures on amplitude integrated EEG (aEEG), and level 3 included other clinical events suggestive of epileptic seizures other than focal clonic or tonic. The clinical events not meeting case definitions (level 4) and those not having an EEG correlate (level 5) were taken as non-seizure events.^4^^,^^18^ Seizure semiology was classified according to the ILAE seizure classification.^18^ The EEG background was grouped according to the following criteria: normal (continuous activity with age appropriate graphoelements and well defined sleep wake cycling); mildly abnormal (continuous activity with mild asymmetry, voltage depression and/or poorly defined sleep wake cycle); moderately abnormal (discontinuous activity with interburst intervals less than 10 s, absent sleep wake cycles and clear asymmetry or asynchrony); severe (discontinuous activity with prolonged interburst intervals more than 10 s, severe attenuation, burst suppression and isoelectric patterns); or undetermined (difficult to assess background due to status epilepticus or excessive artefacts).^19^ Status epilepticus was defined as a seizure burden of 30 min per hour or more in at least one 1-h epoch of EEG recording.^20^ Seizure burden (minutes/hour) was defined as the total duration of ictal discharges (minutes) divided by the total duration of EEG (hours).

All neonates had detailed clinical assessments, electrolyte and blood sugar measurements, infection screening and magnetic resonance imaging prior to hospital discharge. Additional metabolic and genetic investigations were performed as clinically indicated.

### Outcomes

The primary outcome was the onset of seizure freedom within 40 min from of the start of the initial dose of levetiracetam or phenytoin infusion. Seizure freedom was defined as a complete absence of seizures on continuous EEG monitoring for at least 60 min from the end of the last seizure without the need for any additional ASM.

### Statistical analysis

The efficacy of levetiracetam and phenytoin reported as proportions of neonates achieving seizure freedom along with their Clopper Pearson exact 95% confidence limits. To show the time to achieve seizure freedom (endpoint), Kaplan–Meier survival plots are plotted separately for neonates who received levetiracetam as second line ASM, and for neonates who received levetiracetam as well as third line ASM, phenytoin. Data were analysed using SPSS software, version 29.0.

### Role of funding source

This research was funded by the National Institute for Health and Care Research (NIHR) Research and Innovation for Global Health Transformation (NIHR200144) using UK aid from the UK Government to support global health research. The views expressed in this publication are those of the author(s) and not necessarily those of the NIHR or the UK government. The study funders had no role in the study design, data collection, data analysis, data interpretation, or writing of the report. The corresponding author had full access to all the data in the study and had final responsibility for the decision to submit the paper for publication.

## Results

**Fig. 1:**
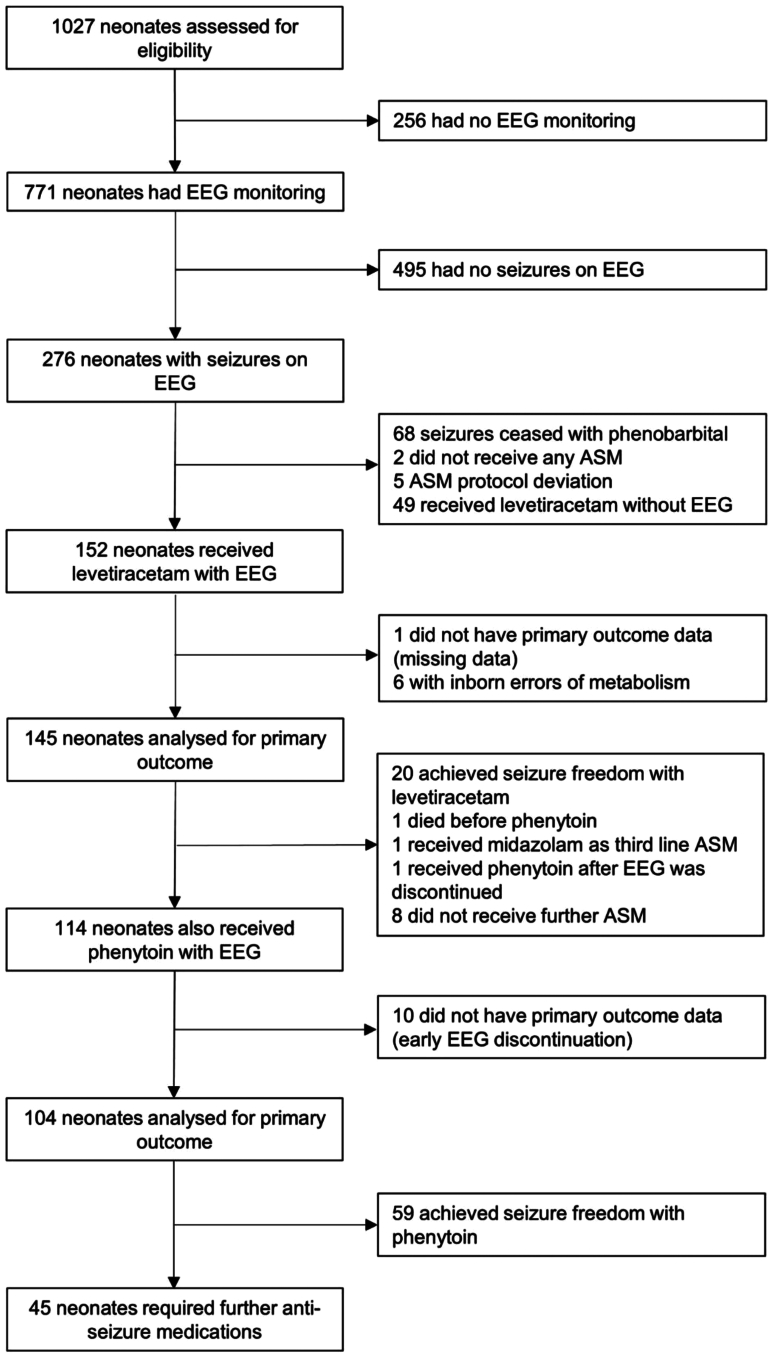
Flow chart of the study. ∗8 neonates had ongoing electrographic seizures after levetiracetam, but the clinicians decided not to administer further ASM as seizure burden was low.

During the 2-year study period, a total of 1027 neonates born at or after 35 weeks were admitted to the neonatal intensive care unit (NICU) with encephalopathy or suspected seizures (Fig. 1). Of these, 771 neonates had EEG monitoring starting at a median (IQR) age of 18.8 (8.5–39.3) hours. A total of 276 of the 771 neonates had EEG confirmed seizures, of which two neonates were not treated with ASM and 68 neonates (25.5%) had seizure termination with phenobarbital and did not require further ASM. Of the 206 neonates who had persistent seizures after phenobarbital, 5 had second line ASM protocol deviations and 49 did not have EEG during second line levetiracetam administration (Supplementary Table S1). The remaining 152 neonates received levetiracetam as second line ASM under EEG monitoring and were enrolled to the study (Fig. 1).

The mean (SD) gestational age of these 152 neonates was 38.7 (1.6) weeks, mean (SD) birth weight was 2589 (538) grams, and 88 (57.9%) were male. Seizure etiologies were HIE in 62 (40.8%) and non-HIE in 90 (59.2%) (Table 1). The non-HIE etiologies included stroke in 20, hemorrhage in 4, brain malformation in 2, sepsis in 16, metabolic in 25 (acute metabolic unresponsive to corrections 19, inborn errors of metabolism 6), and unknown causes in 23 neonates. The enrolled neonates received levetiracetam at a median (IQR) post-natal age of 32.9 (18.7–61.6) hours [range (4.1–247.5)].

**Table 1:** Clinical characteristics.

Clinical characteristics	N	HIE (N = 62)	N	Non-HIE (N = 90)	N	Overall (N = 152)
Gestational age, mean (SD), weeks	62	38.8 (1.4)	90	38.7 (1.8)	152	38.7 (1.6)
Birth weight, mean (SD), grams	62	2621 (496)	89	2566 (567)	151	2589 (538)
Male neonates, n (%)	62	35 (56.5%)	90	53 (58.9%)	152	88 (57.9%)
APGAR 5 min, median (IQR)	57	6.0 (5.0–7.0)	86	8.0 (7.0–9.0)	143	7.0 (6.0–8.0)
Inotropic support, n (%)	62	23 (37.1%)	76	11 (14.5%)	138	34 (24.6%)
Invasive ventilation, n (%)	62	36 (58.1%)	76	18 (23.7%)	138	54 (39.1%)
Death before discharge, n (%)	62	26 (41.9%)	90	17 (18.9%)	152	43 (28.2%)
Seizure type^a^, n (%)						
Electrographic only seizures at all time points	60	32 (53.3%)	88	35 (39.8%)	148	67 (45.3%)
Electroclinical seizures at all time points	60	5 (8.3%)	88	17 (19.3%)	148	22 (14.9%)
Electrographic only seizures at one time point and electroclinical seizures at another time point.	60	23 (38.3%)	88	36 (40.9%)	148	59 (39.9%)
Clinical seizure types, n (%)^b^						
Focal clonic	28	8 (28.6%)	53	29 (54.7%)	81	37 (45.7%)
Tonic	28	6 (21.4%)	53	5 (9.4%)	81	11 (13.6%)
Myoclonic	28	5 (17.8%)	53	6 (11.3%)	81	11 (13.6%)
Spasms	28	0 (0.0%)	53	2 (3.8%)	81	2 (2.5%)
Automatisms	28	10 (35.7%)	53	16 (30.2%)	81	26 (32.1%)
Sequential	28	1 (3.6%)	53	1 (1.9%)	81	2 (2.5%)
Autonomic	28	0 (0.0%)	53	2 (3.8%)	81	2 (2.5%)
Age of starting EEG, median (IQR), hours	62	19.7 (11.7–33.9)	90	44.2 (21.7–67.6)	152	29.2 (15.9–58.6)
EEG background abnormality, n (%)						
Normal	62	0 (0.0%)	89	2 (2.2%)	151	2 (1.3%)
Mild abnormality	62	1 (1.6%)	89	22 (24.7%)	151	23 (15.2%)
Moderate abnormality	62	24 (38.7%)	89	48 (53.9%)	151	72 (47.7%)
Severe abnormality	62	31 (50.0%)	89	12 (13.5%)	151	43 (28.5%)
Undetermined	62	6 (9.7%)	89	5 (5.6%)	151	11 (7.3%)
Status epilepticus, n (%)	62	20 (32.2%)	89	27 (30.3%)	151	47 (31.1%)

a Refers to seizure type after phenobarbital, once the neonate entered the study.

b Data are not mutually exclusive, 1 baby may have more than 1 clinical seizure manifestation.

Among the 152 neonates enrolled, seven infants were excluded from efficacy analysis as per study criteria: six neonates with a diagnosis of inborn errors of metabolism and one infant with missing EEG data. Thus, primary outcome data were available in 145 neonates (Fig. 1) and seizure freedom occurred in 20 (13.8%; 95% CI 8.6%–20.5%) neonates following levetiracetam. Among these, 16 (80.0%) neonates responded to an initial 20 mg/kg dose, and further four (20.0%) to an additional 20 mg/kg (total 40 mg/kg). Of the 125 neonates who had persistent seizures after 40 mg/kg of levetiracetam, one infant died, one received midazolam as third line, one received phenytoin after EEG was discontinued and eight did not receive further ASM as seizure burden was considered low by the clinical team. The remaining 114 neonates received phenytoin as the third line ASM under EEG monitoring. Data on primary outcome were available in 104 out of 114 neonates as 10 neonates had early discontinuation of EEG due to clinical or logistic reasons. The primary outcome of seizure freedom occurred in 59 out of these 104 neonates (56.7%; 95% CI 46.7%–66.4%). Among these, 54 (91.5%) neonates responded to an initial 20 mg/kg dose and further five (8.5%) to an additional 20 mg/kg (total 40 mg/kg). The details of EEG monitoring of neonates analysed at ASM administration are given in Table 2 and scenarios of seizure response following administration of levetiracetam and phenytoin are shown in Supplementary Fig. S1.

**Table 2:** EEG characteristics of neonates analysed at antiseizure medication (ASM) administration.

Characteristics	N	Levetiracetam (second line, N = 145)	N	Phenytoin (third line, N = 104)
Age at ASM administration after birth (median (IQR), hours)	145	32.9 (18.7–61.6)	104	37.7 (20.5–66.0)
Age at ASM administration after seizure onset (median (IQR), hours)	145	19.4 (9.2–49.6)	104	22.3 (10.4–54.6)
Total duration of EEG monitoring after start of response (minutes)				
Median (IQR)	20	115.0 (90.0–133.7)	59	77.0 (65.0–105.0)
Range	20	60–235	59	60–271
Total duration of EEG monitoring to start of next ASM in case of no response (minutes)				
Median (IQR)	125	88.0 (60.0–120.0)	45	120.0 (88.0–150.0)
Range	125	23–435	45	30–520
Seizure type before administration of study ASM (n (%))				
Electrographic only seizures at all time points	140	85 (60.7%)	99	70 (70.7%)
Electroclinical seizures at all time points	140	24 (17.1%)	99	11 (11.1%)
Electrographic only seizures at one time point and electroclinical seizures at another time point.	140	31 (22.1%)	99	18 (18.2%)
Seizure burden before administration of study ASM (median (IQR), minutes/hour)^a^	145	12.0 (4.0–25.0)	104	15.0 (9.0–27.0)
Status epilepticus at administration (n (%))	145	31 (21.4%)	104	19 (18.3%)

a Seizure burden calculated for the entire pretreatment period and was defined as the total duration of seizures in minutes divided by the no. of seizure hours (minutes/hour).

**Fig. 2:**
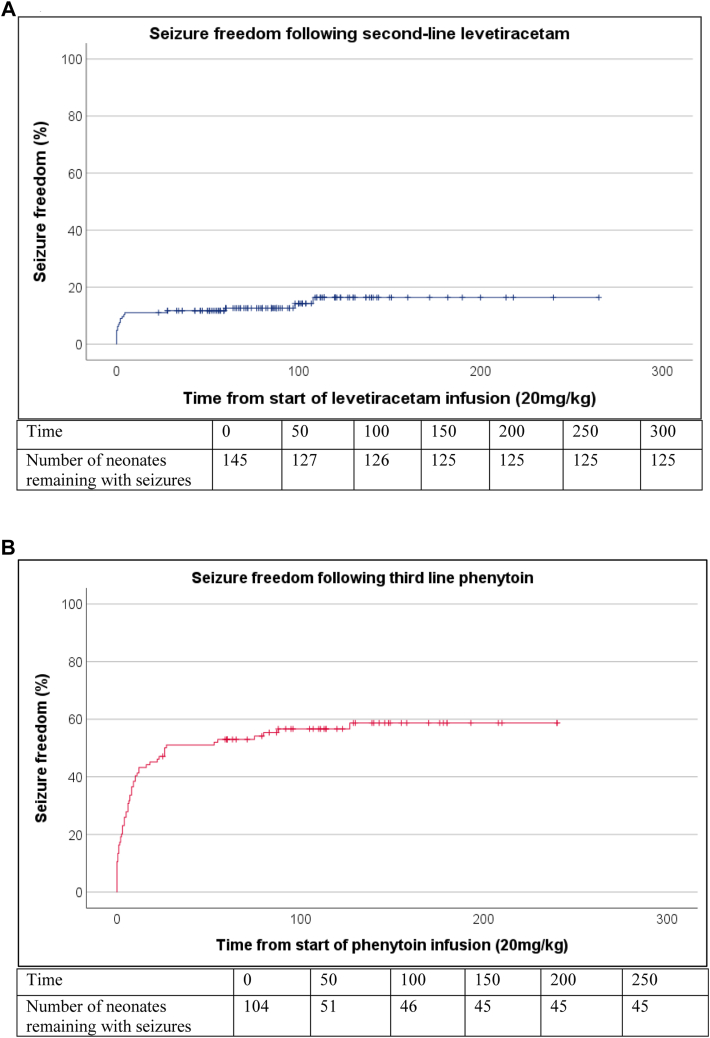
Seizure freedom after second line levetiracetam versus third line phenytoin. Kaplan–Meier plots showing the proportion of neonates with seizure freedom (endpoint, Y-axis) against time (minutes) from the start of initial levetiracetam 20 mg/kg infusion (X-axis) for second line levetiracetam (blue, 3A) and for neonates who also received third-line phenytoin (red, 3B). Note: The Kaplan–Meier estimates of seizure freedom are different to a simple percentage of babies with seizure freedom, as the follow-up time for babies without seizure freedom varied from 25 min to over 3 h. Neonates were censored when they reached endpoint or when the EEG monitoring stopped.

The median (IQR) time gap between the start of infusion of levetiracetam (20 mg/kg) and start of phenytoin was 87.0 (59.5–115.5) minutes and time gap between the start of maximal dose of levetiracetam (40 mg/kg) and phenytoin was 43.0 (30.0–59.5) minutes. The proportion of neonates who reached the efficacy endpoint (primary outcome) was greater with phenytoin as third line ASM [59/104 (56.7%; 95% CI 0.47–0.66)] compared to levetiracetam as second line ASM 20/145 [(13.8%; 95% CI 0.08–0.20)]. The attainment of seizure freedom (endpoint) with time after start of ASM is shown in Fig. 2.

**Fig. 3:**
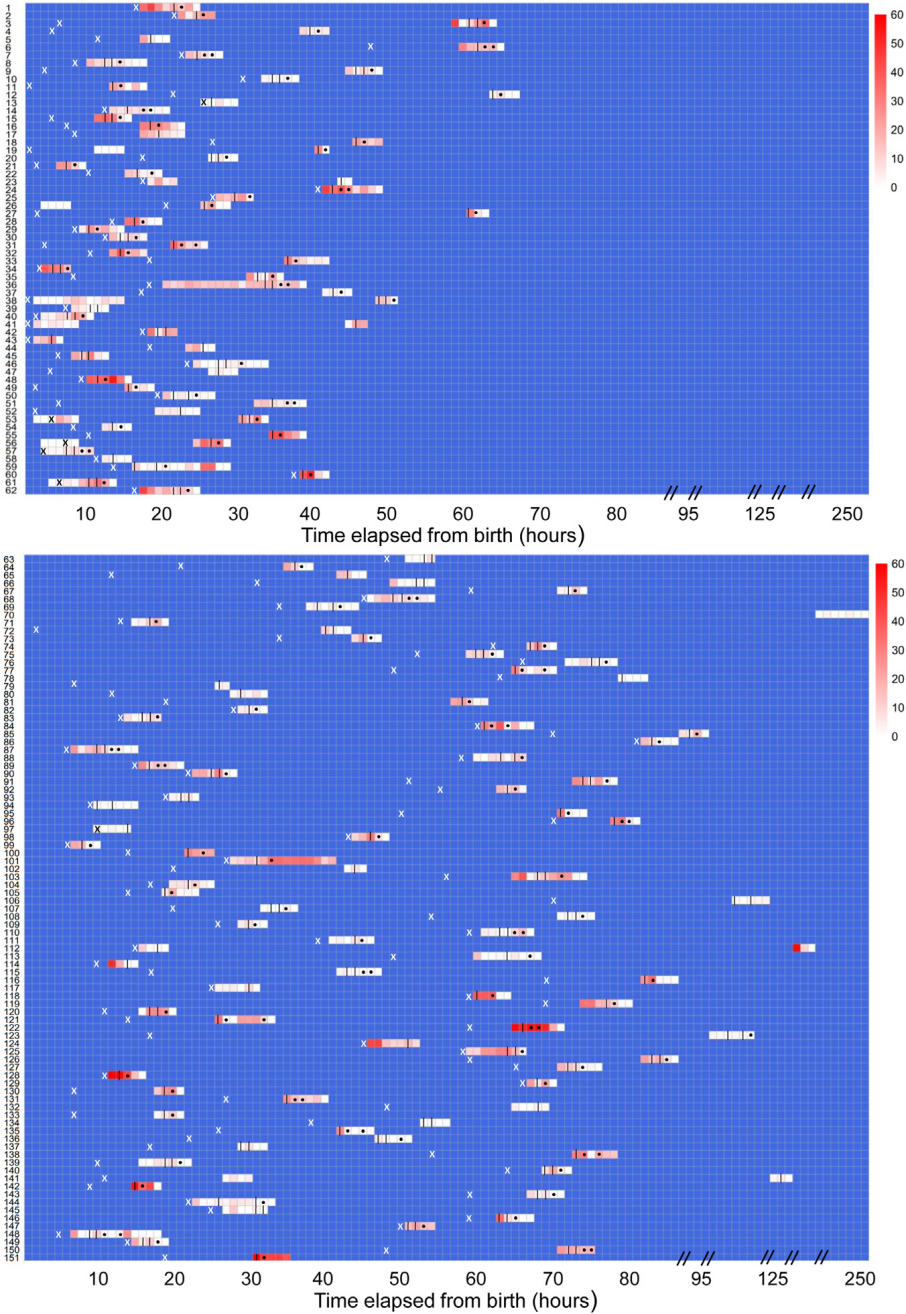
Seizure burden evolution over time. “**|**” = Levetiracetam, “**•**” = Phenytoin, “**X**” = age of onset of seizure (clinical or EEG), Upper panel (3A) shows the seizure burden evolution in neonates with HIE (n = 66) and lower panel (3B) shows non-HIE etiologies (n = 75). X axis- Time elapsed from the birth of baby in hours, Y axis–Each row denotes the evolution of seizure burden in a baby. Seizure burden is represented in minutes per hour and colour coded from white (0 min/h, minimum) to red (60 min/h, maximum). Blue areas represent areas where EEG monitoring was not done. The vertical black line (levetiracetam) and black dot (phenytoin) are points at which each ASM was administered, if two doses were given more than an hour apart, discrete lines are shown.

Details of seizure types and background abnormalities are provided in Table 1. Of the 152 neonates enrolled, EEG data from one neonate was missing. Forty-seven (31.1%) of the remaining 151 neonates analysed had status epilepticus at any point during EEG monitoring. Of these, 31 (21.4%) out of 145 neonates analysed for levetiracetam response were in status epilepticus at levetiracetam administration and 19 (18.3%) out of 104 neonates analysed for phenytoin response were in status epilepticus (Table 2) at phenytoin administration. Among the babies who had status epilepticus, none responded to levetiracetam (0%) whereas 2/19 (10.5%) neonates who were in status during phenytoin administration responded to phenytoin. The baseline median (IQR) seizure burden at the time of levetiracetam administration was 12.0 (4.0–25.0) minutes per hour and at the time of phenytoin administration was 15.0 (9.0–27.0) minutes per hour (Table 2). The evolution of seizure burden over time and with administration of ASM is shown in Fig. 3.

Detailed clinical seizure subclassification was performed on 148 of the 152 recruited neonates where video EEG and continuous vital sign monitoring data were available (Table 1). Of these, 67 (45.3%) were electrographic-only, 22 (14.9%) were electroclinical, while 59 (39.9%) neonates had both electrographic-only and electroclinical seizures at different time points. Among the 81 (54.7%) neonates with electroclinical seizures, focal clonic seizures (45.7%) were the most common clinical manifestation followed by automatisms (32.1%). Of these 81 neonates, 58 were having electro-clinical seizures at the time of levetiracetam administration. Among these 58 neonates, 10 (17.2%) stopped seizing clinically but continued to have electrographic seizures (uncoupling) following the infusion. None of the neonates had serious adverse events like cardiac arrhythmia or sudden cardiac arrest during the time of infusion of levetiracetam or phenytoin.

## Discussion

Here we report, to the best of our knowledge, the largest prospective multicenter study in the world to assess electrographic response to ASM in neonatal seizures using standardized EEG acquisition and analysis protocols, and the first study from an LMIC. Levetiracetam (40 mg/kg) was associated with EEG seizure cessation within 40 min of administration in only 14% of phenobarbital unresponsive neonatal seizures. Additional treatment with phenytoin along with levetiracetam resulted in seizure freedom in further 57% of the neonates. The increase in seizure freedom may be directly related to phenytoin, delayed effects of levetiracetam or synergy. The data presented here will inform future randomised controlled trials of ASM for neonatal seizures in LMIC.

While there are no prospective studies evaluating efficacy of second line levetiracetam on EEG seizure freedom, efficacy data are available from few retrospective studies or subgroups of randomised controlled trials (RCT) comparing first line ASMs. In a retrospective study involving 14 neonates who had persistent seizures despite phenobarbital, Abend et al. reported that four (28%) attained complete seizure freedom.^21^ In a subgroup analysis of a randomised controlled trial (Neolev-2) comparing first line phenobarbital and levetiracetam, six neonates had persistent seizures despite phenobarbital. Of these only one neonate (17%) had complete seizure freedom following levetriracetam.^16^ Although the number of neonates in these studies are too small to draw any meaningful conclusions, the low efficacy of second line levetiracetam for achieving seizure freedom on EEG is consistent with the observations in our study.

Only two studies, both conducted over two decades ago, have reported electrographic response to phenytoin as a second line ASM. First was a landmark RCT comparing phenobarbital and phenytoin that included a subgroup of 15 neonates treated with phenytoin for non-response to phenobarbital; seizure freedom on EEG occurred in 4 (27%).^22^ Another was a prospective observational study that included six neonates who had persistent seizures after phenobarbital; seizure freedom on EEG was not achieved in any of these six neonates. However, the number of neonates in these studies is too small to make meaningful comparisons about the efficacy of second line phenytoin treatment.^23^

In contrast, open label studies without EEG monitoring have reported much higher efficacy of levetiracetam (71%–93%)^24^^,^^25^ in termination of clinical seizures. This may be related to observer bias inherent in open label interventions, subjectivity in the diagnosis of neonatal seizures and possibly, electroclinical uncoupling.^26^^,^^27^ Hence pilot randomised controlled trials comparing different ASM,^13^ where the investigators are neither masked to the intervention nor the outcome, are prone to serious bias.^27^ In our study only 10 (17%) neonates with electroclinical seizures had uncoupling to electrographic-only seizures after administration of levetiracetam.

The critical importance of EEG in the evaluation of ASM efficacy is highlighted by the contrasting results of RCTs using clinical or EEG seizure freedom as primary endpoints. An open label RCT trial involving 100 neonates reported that first line levetiracetam was superior to phenobarbital in clinical seizures cessation (86% versus 62%; p < 0.01),^28^ while a blinded RCT involving 83 neonates reported that phenobarbital was superior to levetiracetam (80% versus 28%; p < 0.001) in achieving seizure cessation on EEG despite the latter trial using a higher dose of levetiracetam of up to 60 mg/kg.^16^

It is important to note that none of the RCTs of ASM for neonatal seizures have reported neurodevelopmental outcome at 18 months or more.^29^ A less effective ASM that leads to a better neurodevelopmental outcome is preferable to a highly effective ASM that adversely affects the neurodevelopment. In the original National Institute of Human Development and Child Health Neonatal Research Network hypothermia trial,^30^ ASM medications were associated with adverse outcomes after HIE.^31^ The confounding effects of the underlying brain injury, neonatal seizures and ASM on neurodevelopment, can be examined only in carefully designed and adequately powered double blind RCTs using EEG and robust neurodevelopmental outcome evaluation.

The main strength of our study is the large number of neonates we were able to enrol with continuous video EEG monitoring, particularly in a LMIC setting. We also had trained neonatal neurology fellows and technicians to allow real time EEG reporting and feedback to the clinical team. We carefully annotated the ASM start points on the EEG which enabled us to accurately quantify measures such as seizure burden before and after the administration of the ASM. Building on this work, we have established a Collaborative Neonatal Neuroprotection Trial platform in South Asia (CONNECTIONS) to conduct large multi-country trials of ASM and other neuroprotective therapies.

Our study had several limitations. Firstly, our study design was observational and direct comparisons cannot be made as additional confounders and temporal changes may have influenced the efficacy of ASM unequally. Thus, neonates in levetiracetam group had both phenobarbital and levetiracetam, while those in the phenytoin group had phenobarbital, levetiracetam and phenytoin. Therefore, synergy or later effect of levetiracetam could have amplified the efficacy of phenytoin. Nevertheless, poor (14%) seizure cessation even after 40 min of levetiracetam administration is a concern, particularly as 31% of the neonates were in status epilepticus in our study. Furthermore, neonatal seizures tend to be more refractory to treatment over time.^32^ Despite this phenytoin as a third line was associated with more seizure freedom than second line levetiracetam. Although seizures in HIE tend to peak around 24 h before naturally decreasing by 72 h, the median time interval between full dose of levetiracetam and phenytoin was too short (43 min) for these temporal changes to modify the treatment efficacy in our study.

Secondly, although our study protocol required the levetiracetam infusion to be completed within 30 min, we did not collect the exact time when the infusion was completed. To account for any potential delays, we used seizure freedom within 40 min from the start of the infusion.

Third, we used a maximal dose of 40 mg/kg of levetiracetam as safety data on high dose levetiracetam are lacking, and hence cannot exclude a better efficacy with higher doses.^33^

Fourth, our primary outcome was based on seizure freedom within 40 min of the administration and not 24 h as in earlier studies from high-income countries.^16^ Temporary cessation of seizures is frequent after ASM is given.^34^ Hence in clinical trials, EEG monitoring is recommended to continue for at least 24 h after seizure cessation to detect any possible recrudescence of seizures, and because seizures frequently wax and wane over hours. However, acquiring continuous EEG monitoring over 24 h was logistically challenging as electrical and movement artefacts were common in Indian neonatal units. Hence EEG technicians or neonatal fellows trained in neonatal EEG had to be present at the bedside to ensure data quality in our study.

Finally, whole body-hypothermia was being offered at the participating sites before the publication a randomised controlled trial (Hypothermia for Encephalopathy in Low and Middle-Income countries; HELIX)^35^ reporting lack of neuroprotection and increased mortality with this treatment, and hence was de-implemented. Thus, only one neonate received whole-body hypothermia in this study and the body temperature of other neonates was maintained in the normothermic range. Whole-body hypothermia may reduce seizure burden, and lower the renal clearance of levetiracetam,^36^^,^^37^ all of which may modify the treatment response. The disease severity among neonates recruited to our study was also high compared to high-income countries, as observed by the high rates of status epilepticus and mortality before discharge. Therefore, the results may not be generalisable to neonates in high-income countries or those treated with whole-body hypothermia.

In this large multicenter observational study involving neonates with seizures unresponsive to phenobarbital, levetiracetam at a maximal dose of 40 mg/kg, was associated with attainment of seizure freedom on EEG only in 14% of the neonates by 40 min after the start of the infusion. As 31% of the neonates were in status epilepticus, low efficacy of levetiracetam in EEG seizure cessation is of concern. Additional treatment with phenytoin along with levetiracetam resulted in seizure freedom in further 57% of the neonates. The increase in seizure freedom may be directly related to phenytoin, delayed effects of levetiracetam or synergy.

## Contributors

VK recruited babies, interpreted the EEG data and wrote the first draft under supervision of RP and ST. VK, VU, HV, MKA, and PW recruited babies, interpreted the EEG data. SF, SC, SD, MK, VKG, and AVT supervised the site recruitments. PMo, CB, RG, PM and SP assisted in trial management and preparation of the manuscript. CRN, JHC, SS assisted in protocol development, interpretation of the data and preparation of the manuscript. PB was responsible for all statistical analysis. RP assisted in protocol development, reviewed all EEGs and was responsible for the interpretation and analysis. All authors approved the final version of the manuscript. ST obtained funding, supervised all aspects of the study including data analysis and interpretation, preparation of the manuscript and had final responsibility for the decision to submit for publication.

## Data sharing statement

Anonymised participant data used in this study will be available from the corresponding author after approval of a proposal with a signed data access agreement.

## Declaration of interests

Helen Cross has received institutional renumeration from Zogenix, Union Chimique Belge (UCB), Marinius, Stroke Therapeutics, Ultragenyx, GW Pharma, Jazz, Biocodex for educational symposium and advisory board activities and renumeration for administrative support from International League Against Epilepsy at the President. Ronit Pressler has received institutional funding from UCB and personal renumeration from Kephala and Natus for lectures. The authors declare no conflict of interest.
