# Supplementary material for: Sequential levetiracetam and phenytoin in electroencephalographic neonatal seizures unresponsive to phenobarbital: a multicenter prospective observational study in India
Source: Lancet Reg Health Southeast Asia. 2024 Feb 15;25:100371. doi: 10.1016/j.lansea.2024.100371 (PMC467079; doi:10.1016/j.lansea.2024.100371)
Supplement: Supplementary Table S1 [file mmc1.docx]

**Supplementary table 1: Clinical characteristics and EEG monitoring data of excluded babies.**

| **Characteristics** | **N** | **n** |
| --- | --- | --- |
| Gestational age (weeks, mean (SD)) | 54 | 38.3 (1.9) |
| Birth weight (grams, mean (SD)) | 54 | 2742 (576) |
| APGAR 5 minutes (median (IQR)) | 53 | 7.00 (6.00-8.00) |
| Male neonates (n (%)) | 54 | 34 (62.9%) |
| Age at start of EEG (hours, median (IQR)) | 54 | 24.5 (12.1-48.1) |
| Age at start of EEG (range) | 54 | 1.4-83.0 |
| Duration of EEG recording (hours, median (IQR)) | 54 | 4.0 (4.0-5.1) |
| Age at seizure onset (clinical or EEG) (hours, median (IQR)) | 54 | 17.0 (7.0-51.0) |
| Age at identification of seizure on EEG (hours, median (IQR)) | 54 | 29.0 (17.2-52.0) |
| Causes of seizure (n (%)) | 54 |  |
| - Hypoxic ischemic encephalopathy |  | 24 (44.4%) |
| - Stroke |  | 4 (7.4%) |
| - Infection |  | 4 (7.4%) |
| - Metabolic |  | 5 (9.2%) |
| - Malformations |  | 1 (1.8%) |
| - Inborn errors of metabolism |  | 4 (7.4%) |
| - Suspected genetic/epilepsy syndrome |  | 1 (1.8%) |
| - Unknown |  | 11 (20.4%) |
| Death before discharge (n (%)) | 54 | 13 (24.1%) |
