# Supplementary material for: Sequential levetiracetam and phenytoin in electroencephalographic neonatal seizures unresponsive to phenobarbital: a multicenter prospective observational study in India
Source: Lancet Reg Health Southeast Asia. 2024 Feb 15;25:100371. doi: 10.1016/j.lansea.2024.100371 (PMC467079; doi:10.1016/j.lansea.2024.100371)
Supplement: Supplementary Fig. S1 [file mmc2.docx]

**Supplementary figure 1: Examples of seizure response after levetiracetam and phenytoin.**

**
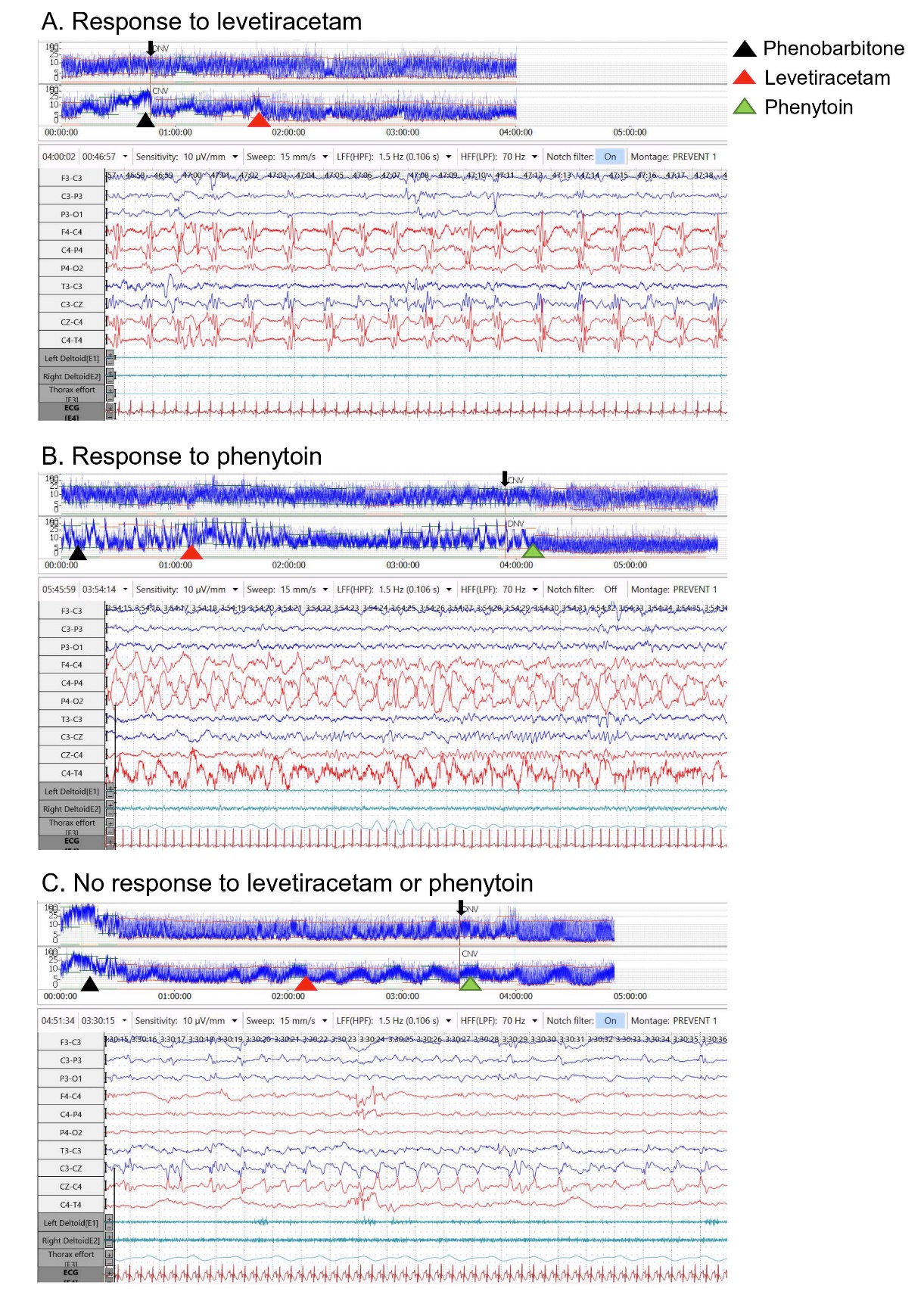
**

Legend: Black downward pointing arrow on aEEG represents the timepoint of seizure shown in the EEG trace. Arrows pointing upwards represent antiseizure medications (black=phenobarbital, red=levetiracetam and green=phenytoin). Figure 2A shows an example of seizure freedom following levetiracetam administration; 2B shows an example of seizure freedom following phenytoin administration; and 2C shows an example where the neonate did not respond to either levetiracetam or phenytoin.
